# Prevalence and risk factors of tungiasis among children of Wensho district, southern Ethiopia

**DOI:** 10.1186/s12879-018-3373-5

**Published:** 2018-09-10

**Authors:** Mekonnen Girma, Ayalew Astatkie, Solomon Asnake

**Affiliations:** 1 School of Medicine, College of Medicine and Health Sciences, Hawassa University, Hawassa, Ethiopia; 2 School of Public and Environmental Health, College of Medicine and Health Sciences, Hawassa University, Hawassa, Ethiopia

**Keywords:** Tungiasis, Prevalence, Risk factors, Ethiopia

## Abstract

**Background:**

Tungiasis is an ectoparasitic infestation, which still has public health importance in deprived populations of developing countries. Data on the prevalence and risk factors of tungiasis is rare in Ethiopia. Hence, this study was designed to determine the prevalence and risk factors of tungiasis among children in Wensho district, southern Ethiopia.

**Methods:**

From February to May 2016, we conducted a community-based cross-sectional study on 366 children 5–14 years old. Data about the presence and severity of tungiasis were obtained through inspection and data on risk factors were collected through interviews of parents/guardians of the children using structured questionnaire and through observation of the housing environment using structured checklist.

**Results:**

Two hundred fifteen (58.7%, 95% confidence interval [CI]: 53.7%, 63.8%) of the 366 children were infested with *Tunga penetrans*. Most lesions were localized in the feet and the distribution of the disease by sex was similar (57.4% among males and 60.3% among females). Children of illiterate mothers (adjusted odds ratio [AOR]: 3.62, 95% CI: 1.35, 9.73) and children whose mothers have attended only primary education (AOR: 2.72, 95% CI: 1.06, 6.97), children from cat owning households (AOR: 4.95, 95% CI: 1.19, 20.60) and children who occasionally use footwear (AOR: 7.42, 95% CI: 4.29, 12.83) and those who never use footwear (AOR: 12.55, 95% CI: 3.38, 46.58) had a significantly higher odds of tungiasis infestation.

**Conclusion:**

Tungiasis is an important public health problem with considerable morbidity among children in Wensho. Hence, implementation of tungiasis prevention strategies such as promoting shoes wearing, provision of health education, fumigating the residential houses and applying insecticides on pets are recommended.

## Background

Tungiasis, an infestation caused by *Tunga penetrans* (sand flea or jigger flea), is common in poor communities of the tropical and subtropical part of the world [1]. The Periungual region of the toes is the most preferred site by the flea, although infestation can also occur in hands, elbows, and genital and anal regions [2]. The infestation is more prevalent in children, particularly among 5–10 years old children than in adults [3]. Living with reservoir domestic animals such as cats, dogs and pigs [4], poor personal hygiene, poor sanitation of the housing and residential environment, and lack of foot wear [5–7] are risk factors associated with tungiasis infestation.

Common sequelae associated with Tungiasis are acute and chronic inflammation of toes, fissures, lymphoedema and deformation and loss of toe nails. Severe inflammation, ulceration and fibrosis, and though rarely, death might occur in case of profound infestation [8]. Locally, people extract the mature flea using sterile needle. Moreover, topical application of dimeticones of low viscosity can execute entrenched sand fleas effectively [9]. As a sub-Saharan African country, though infestation is common in Ethiopia, published data on the prevalence and risk factors of tungiasis are scant; only a few studies have reported on the prevalence and/or risk factors of tungiasis in Ethiopia [5, 10, 11]. Hence, this study was designed to determine the prevalence of tungiasis and associated risk factors among children in Wensho district, southern Ethiopia.

## Methods

### Study area

**Fig. 1:**
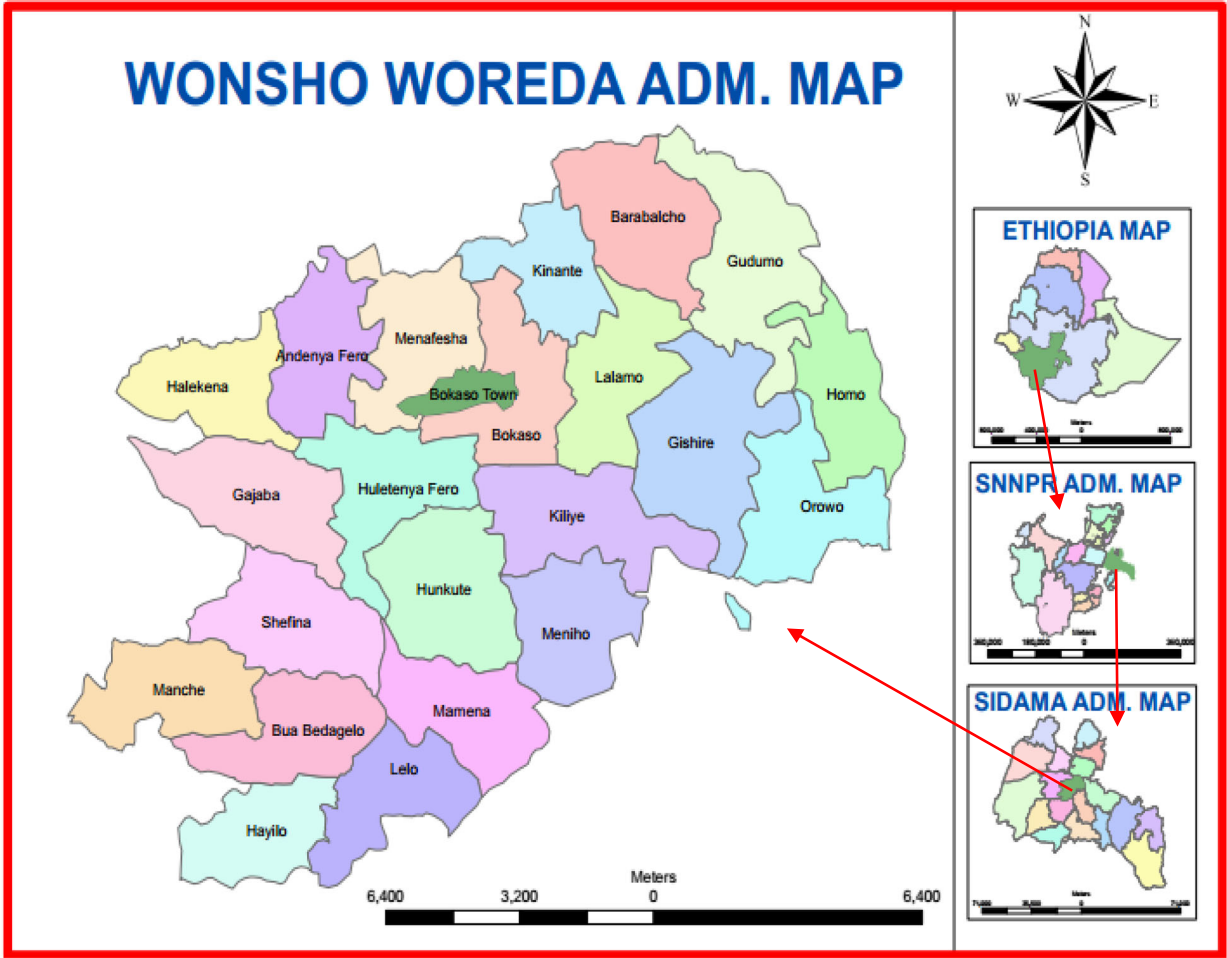
Map of Wensho district (source: Sidama Zone Health Department, Southern Ethiopia). SNNPR: Southern Nations Nationalities and Peoples Region, ADM: Administration

The map of the district is shown in Fig. 1. Wensho is one of the districts located in the Sidama zone of the Southern Nations, Nationalities, and Peoples Region (SNNPR) of Ethiopia. The district has 23 *kebeles* (lower administrative units in Ethiopia) of which only one is urban. Based on the 2007 population Census of Ethiopia, the district has a total population of 89,662 with a male-to-female ratio of 1.03. Only 2.27% of its population resides in an urban area (Personal communication: Wensho District Health Office, 2016).

### Study design and population

This was a community-based cross-sectional study conducted among children 5–14 years old permanently residing in Wensho district. The study was limited to children five years and older because they are the most vulnerable group [12].

### Sample size and sampling procedure

The sample size required both for estimating prevalence and determining risk factors was calculated under different assumptions using the software Epi Info version 7. Finally, the sample size (*n* = 366) estimated for estimating the prevalence of tungiasis with the assumptions of an expected prevalence of 34.7% based on a study by Walker et al. [11], a 95% level of confidence, a 5% margin of error and an anticipated nonresponse rate of 5% was found to be the largest and sufficient for both objectives.

Of the twenty-three kebeles in Wensho district, five kebeles, namely, Andenya Fero, Huletenya Fero, Gejaba, Haleqena and Hunqute were purposively selected for inclusion into the study due to their accessibility. From each selected kebele, every fourth household was selected for the study. In each selected household, a child 5–14 years old available at the time of the visit was included in the study. If two or more 5–14 years old children were available at the time of the visit, one was selected at random. If the selected household doesn’t have a child 5–14 years old, the next household was selected.

### Data collection

Data were collected by Health Extension Workers (HEWs) after a thorough training about the data collection tools and procedures. The data collection involved three methods: physical examination of the children, interview of parents/guardians of the children and observation of the home environment of the children. Physical examination of the children was done for the presence of embedded tungiasis in legs, feet, hands and arms. At the examination, the findings were considered diagnostic for parasitologcal characteristics of jigger infestation based on the Fortaleza classification: from a dark and itching spot in the skin to a characteristic crater-like sores in the skin or suppurative lesions in natural history of disease [13]. The number of lesions and embedded sites was documented. A mild infestation was defined as the presence of one to five lesions, a moderate infestation as the presence of six to thirty lesions, and heavy infestation as the presence of > 30 lesions [6, 7].

The parents/guardians of the children were interviewed using a structured questionnaire comprising demographic, socio-economic, environmental and behavioral variables along with disease-associated conditions. Sanitary and other pertinent conditions of the housing environment were assessed through observation using structured checklist.

### Data analysis

All data from the questionnaire and examination were entered, edited, and analyzed using IBM SPSS version 20.0 (Armonk, NY: IBM Corp). Descriptive analysis was done to compute proportions for describing the basic characteristics of the studied children and the prevalence of tungiasis. Binary logistic regression was conducted to identify the risk factors of tungiasis. The logistic regression analysis started with a crude analysis whereby each possible determinant was individually investigated for an association with tungiasis. Variables found to have *p*-values up to 0.25 in the crude analysis and those deemed important based on previous studies were included into the initial multivariable model. A series of model refinement and refitting were done until a more plausible and fit model was arrived at. Adjusted odds ratios (AORs) with 95% confidence intervals (CIs) were used to judge the presence and strength of association between tungiasis and the different risk factors. AORs not embracing 1 were considered statistically significant.

### Ethical consideration

The study was conducted after obtaining ethical approval by the Institutional Review Board at the College of Medicine and Health Sciences of Hawassa University, Hawassa, Ethiopia. Different administrative officials of the study site also provided permission to conduct the study in the community. Each child was enrolled in the study after the parent/guardian signed a consent form. Besides, each child gave an assent before taking part in the study. At the end of the study, each child was given a bar of soap for personal hygiene and a needle, a surgical blade and Vaseline ointment to help them remove the flea if and when they get infested.

## Results

All the proposed 366 study participants took part in the study. The study participant were 5–14 years old with a mean (± standard deviation) age of 8 (± 2.0) years. About three-fourth (74%) were attending school from kindergarten to grade six. Spring or river was the major source of water used by majority (79.9%) of the participants. When asked of methods used to remove jigger fleas from affected body parts, 99.2% of the children’s parents/guardians reported extraction using needle or thorns and 0.8% reported using chemicals (Table 1).

**Table 1:** Characteristics of the study participants, Wensho district, southern Ethiopia, 2016

Variable	Number (*n* = 366)	Percentage
Age category		
5–9	281	76.8
10–14	85	23.2
Sex		
Male	195	53.3
Female	171	46.7
Schooling of the child		
Not attending school	94	25.7
Attending school	272	74.3
Family size		
≤ 5	201	54.9
6–10	162	44.3
> 10	3	0.8
Educational status of the mother		
Not educated	160	43.7
Primary school	170	46.4
Secondary school and above	36	9.8
Educational status of the father		
Not educated	37	10.1
Primary school	166	45.4
Secondary school and above	163	44.5
Source of household water supply		
Pipeline/tap water	74	20.2
Well/bore hole	7	1.9
Spring/river	285	77.9
Household waste disposal system		
Public collection	185	50.5
Thrown on compound	9	2.5
Burned and buried	172	47.0
Methods used to remove fleas from the body		
Use of chemicals/natural products/black oil	3	0.8
Remove by thorns	103	28.1
Remove by needle/pin	260	71.0

**Fig. 2:**
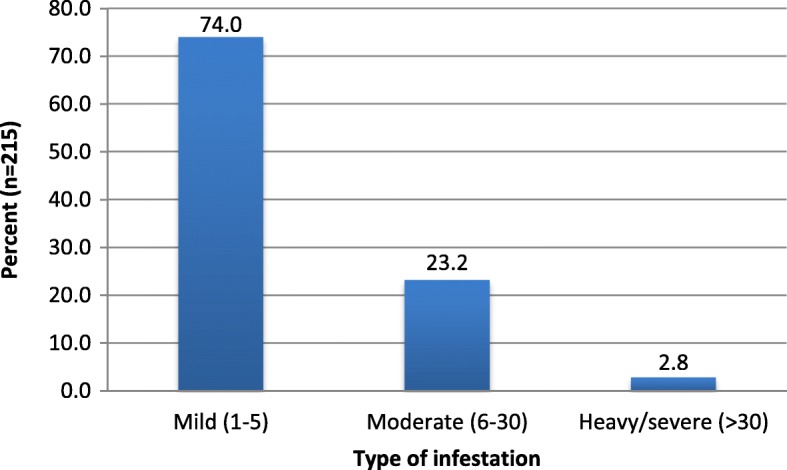
Type of infestation among children with tungiasis, Wensho district, Southern Ethiopia, 2016

Two hundred and fifteen of the 366 children were found to have tungiasis, thus making the prevalence of tungiasis among children in Wensho to be 58.7% (95% CI: 53.7%, 63.8%). The prevalence among males was 57.7% whereas among females it was 60.3%. In 208 (96.7%) of the children with tungiasis, the lesions were located on the feet; however, 12(5.6%) of the children presented with lesions on the hands. A total of 1464 lesions were found on 215 cases with a mean parasite intensity of 6.8 jiggers per a child. Most (74%) of the children with tungiasis had mild infestation, while 50(23.3%) had moderate and 6(2.8%) had heavy/sever infestation (Fig. 2). Most common symptoms among those who were infested were ulcer (70.2%), loss of nails (34%) and deformation of toe or fingers (27.4%) and suppuration (26%).

Different factors were investigated for possible association with tungiasis infestation. These factors included age of the child, sex of the child, educational status of the mother, educational status of the father, family monthly income, floor material of the house in which the children live, types of domestic animals reared by the household (dogs and cats), presence of rats, source of water supply, waste disposal system and use of footwear. After exploring the association of these factors with tungiasis infestation using unadjusted (bivariate) logistic regression model, maternal educational status, floor material of the house, presence of cats in the household, use of footwear and water source were included in the multiple logistic regression model.

Accordingly, maternal educational status, use of footwear and presence of cats in the house were found to be significantly associated with tungiasis infestation of children. Compared to children of mothers with secondary education and above, children of illiterate mothers were found to have 3.62 times higher odds of tungiasis infestation (AOR: 3.62, 95% CI: 1.35, 9.73) and those from mothers with primary education were found to have 2.72 times higher odds of tungiasis infestation (AOR: 2.72, 95% CI: 1.06, 6.97). Children from cat owning households were also found to have 4.95 times higher odds of tungiasis infestation (AOR: 4.95, 95% CI: 1.19, 20.60). Besides, compared to children who always use footwear, children who occasionally use footwear had 7.42 times higher odds of tungiasis infestation (AOR: 7.42, 95% CI: 4.29, 12.83) and those who seldom or never use footwear had 12.55 times higher odds of tungiasis infestation (AOR: 12.55, 95% CI: 3.38, 46.58) (Table 2).

**Table 2:** Determinants of tungiasis infestation among children in Wensho woreda, southern Ethiopia, 2016

Determinant	Tungiasis infestation		Crude OR (95% CI)	Adjusted OR (95% CI)
	Yes, n (%)	No, n (%)		
Mother’s educational status				
Illiterate	115 (71.9)	45 (28.1)	**7.67 (3.35, 17.57)**	**3.62 (1.35, 9.73)**
Primary	91 (53.5)	79 (46.5)	**3.46 (1.53, 7.79)**	**2.72 (1.06, 6.97)**
Secondary	9 (25.0)	27 (75.0)	1	1
Material of the floor of the house				
Earthen	125 (72.3)	48 (27.7)	**4.69 (2.30, 9.57)**	1.81 (0.76, 4.31)
Bamboo	75 (49.7)	76 (50.3)	1.78 (0.88, 3.60)	1.34 (0.59, 3.06)
Cement	15 (35.7)	27 (64.3)	1	1
Household source of water				
Pipeline	36 (48.6)	38 (51.4)	1	1
Well/ bore hole	4 (57.1)	3 (42.9)	1.41 (0.29, 6.73)	0.62 (0.10, 4.09)
Spring/river	175 (61.4)	110 (38.6)	1.68 (1.0, 2.81)	0.60 (0.32, 1.14)
Presence of cat in the house				
Yes	11 (78.6)	3 (21.4)	**2.66 (0.73, 9.70)**	**4.95 (1.19, 20.60)**
No	204 (58.0)	148 (42.0)	1	1
Use footwear				
Always	55 (32.7)	113 (67.3)	1	1
Occasionally	138 (79.8)	35 (20.2)	**8.10 (4.96, 13.24)**	**7.42 (4.29, 12.83)**
Never	22 (88.0)	3 (12.0)	**15.07 (4.32, 52.51)**	**12.55 (3.38, 46.58)**

*OR* odds ratio, *CI* confidence interval; odds ratios showing statistically significant association are presented in bold typeface

## Discussion

This study found a high (58.7%) prevalence of tungiasis in Wensho district, a rural setting, in southern Ethiopia. Significantly higher odds of tungiasis were found among children whose mothers were illiterate or had primary school education compared to children whose mothers had attended secondary education. Children from households owning cats also had higher odds of tungiasis infestation. Furthermore, children who never or rarely use footwear had significantly higher odds of tungiasis than children who always use footwear.

The prevalence of 58.7% found by the current study is much higher than the prevalence rate of 1.2% previously reported from a national survey in Ethiopia [10], 34.7% reported from Yirgacheffe district in southern Ethiopia and 1.6% reported from Brazil [14]. The reason for the difference could be that the current study was done in a dry season when the incidence is supposed to be at its peak. On the other hand, the prevalence found by the current study is comparatively equivalent to or lower than the results reported in children of Cameroon, where the prevalence ranged from 60.5 to 70.2% [15], while this rate is extremely lower than the 97% prevalence reported from Northern Tanzania [16]. Climatic, socioeconomic and cultural factors might have contributed to variations in jigger infestation rate of different epidemiological settings [17].

The apparently high prevalence of tungiasis found by the current study implies that tungiasis is a huge problem with important public health ramifications among children in Wensho. The inflammation in the part of the body where the jigger flea burrows is often unbearable and disturbing. Consequently, people are forced to try to get rid of the flea from the body using needle, thorn or other sharp materials. Yet, such materials are often not sterile and may be shared among people and serve as a way of transmitting blood-borne infections such as hepatitis B and C and human immunodeficiency virus (HIV) [18]. The lesion may also be complicated by bacterial superinfection; even tetanus may be causally linked to tungiasis in areas with low immunization coverage [12, 18]. Tungiasis entails chronic sequelae such as chronic pain, disfigurement and mutilation of the feet and impaired mobility [18]. Thus, it adversely affects quality of life [11] and household economy [18]. It also negatively affects the school attendance and performance of children [11, 18].

In the current study, the prevalence of tungiasis did not differ by gender. Prevalence between the sexes may differ from community to community. Differences in the prevalence of tungiasis between males and females reported in various studies in Africa were not statistically significant [6, 16, 17]. However, a study in Cameroon has demonstrated tungiasis to be more prevalent among males than among females [15].

With regard to actions taken when infested, majority of respondents reported mechanical removal using needle or thorns. This finding is consistent with findings from northeast Brazil and Nigeria [8, 19]. This may imply an attempt to evacuate the jigger flea with locally available materials. However, such materials are often not sterile and may be shared among people and hence may cause bacterial superinfection and serve as a way of transmission of blood-borne infection [18]. This may necessitate the provision of health education to at-risk communities regarding how to manage tungiasis.

In most (about 97%) of the children, the lesions were localized on the feet. This is in agreement with results of studies carried out in a rural population of Northwest Cameroon, Lagos State of Nigeria and northeast Brazil [3, 15, 20]. This might be explained by the fact that the jigger flea is a poor jumper so that most lesions or embedments are confined to the feet instead of ectopic sites [3].

Low level of education of the mother was found to be an important predictor of jigger flea infestation of children. A low educational level, and particularly illiteracy, has been shown to be associated with tungiasis infestation in a previous study as well [7]. This may be related to the low economic status of uneducated or less educated mothers whereby they are unable to fulfill necessities such as shoes for their children.

Domestic animal reservoirs such as pigs, dogs and cats have been frequently identified as important for human tungiasis [21]. Studies done in Nigeria and Kenya have reported domestic reservoir animals to be risk factors for occurrence of tungiasis in a community [1, 6, 22]. The present study showed that the ownership of cats by families was associated with tungiasis infestation. On the other hand, studies in north Brazil and Nigeria have respectively shown that ownership of dogs [7] and pigs [6] are significantly associated with tungiasis. These findings correspond with the thought that in the tropics the human and domestic cycles closely overlap [23].

Failure to wear shoes when walking in soil infested with fleas is the foremost attributing factor for contracting tungiasis. Regular wearing of proper footwear may help in preventing or slowing down the progression of many neglected tropical diseases (NTDs) including tungiasis [6, 24, 25]. In line with this, lack of consistent use of footwear emerged to be a very significant factor associated with occurrence of tungiasis in our study.

The results of the present study should be interpreted considering its limitation. The study kebeles included in the present study were selected purposively for their accessibility. Hence, our sample of children may not be representative of all the children in Wensho district. Kebeles that were not easily accessible may have higher risk of tungiasis. Therefore, their exclusion might have resulted in underestimation of the prevalence of tungiasis.

## Conclusion

This study indicated that tungiasis is a common and important public health issue affecting nearly six in ten young children in a rural setting of south Ethiopia. The presence of tungiasis is associated with education status of the mothers, ownership of cat and nonuse or inconsistent use of foot wear by children. Thus, educating heads of households or people about the risk factors, fumigating residential houses, dusting or spraying domestic animals (such as cats) with insecticides and creating awareness on importance of regular use of footwear are needed.

## Abbreviations

AOR: Adjusted odds ratio
CI: Confidence interval
HEWs: Health Extension Workers
HIV: Human immunodeficiency virus
HU: Hawassa University
IRB: Institutional Review Board
NTDs: Neglected tropical diseases
RDD: Research and Development Directorate
SNNPR: Southern Nations, Nationalities and Peoples Regions
*T. penetrans*: *Tunga penetrans*
